# *In Situ* Formation of Steroidal Supramolecular Gels Designed for Drug Release

**DOI:** 10.3390/molecules18043745

**Published:** 2013-03-25

**Authors:** Hana Bunzen, Erkki Kolehmainen

**Affiliations:** Department of Chemistry, University of Jyväskylä, P.O. Box 35, FI-40014, Finland; E-Mail: erkki.t.kolehmainen@jyu.fi

**Keywords:** organogel, acid-responsive, cholesterol, *in situ* gelation, drug release

## Abstract

In this work, a steroidal gelator containing an imine bond was synthesized, and its gelation behavior as well as a sensitivity of its gels towards acids was investigated. It was shown that the gels were acid-responsive, and that the gelator molecules could be prepared either by a conventional synthesis or directly *in situ* during the gel forming process. The gels prepared by both methods were studied and it was found that they had very similar macro- and microscopic properties. Furthermore, the possibility to use the gels as carriers for aromatic drugs such as 5-chloro-8-hydroxyquinoline, pyrazinecarboxamide, and antipyrine was investigated and the prepared two-component gels were studied with regard to their potential applications in drug delivery, particularly in a pH-controlled drug release.

## 1. Introduction

Steroids, as compounds with a wide array of different tasks in Nature, come in many varieties. But all of them combine a large and rigid framework with functional groups making them especially valuable as starting materials for organic synthesis. In this article we want to show the application of steroids in supramolecular gels. Supramolecular gels [1,2,3,4,5,6] are smart functional nanoscale materials with high potential for a wide range of advanced applications, among others [7,8,9,10,11], in drug delivery [12,13,14], tissue engineering [12], light-harvesting systems [7,9,15], and optoelectronics [10,11]. Their formation stems from spontaneous but controlled self-assembly of small molecules, often called low-molecular-weight gelators (LMWGs), into a three-dimensional network with solvent molecules entrapped in its cavities [5,16]. Despite their mostly liquid composition, these systems demonstrate the appearance and rheological behaviour of solids. Besides steroids [17,18,19,20] other examples of supramolecular gels, based on natural building blocks like nucleobases [21,22,23], amino acids, or oligopeptides [24,25,26,27,28], have been reported.

In recent years supramolecular gels have received increased attention as alternative materials to conventional polymer gels, mostly due to the possibility to install desired properties into a gelator structure, *i.e.*, on a molecular level [7]. By a proper design of LMWGs, the self-assembly of the molecules can be arranged and controlled. Appropriately designed gelators, *i.e.*, decorated with suitable functional groups, can be programmed for example to be responsive to external stimuli such as light, mechanical stress, pH, ionic strength, metal ions, or anions [29,30,31,32,33,34]. In most cases, a change on a molecular level results directly in a change of macroscopic properties of the whole system. Recently, several stimuli-responsive steroidal supramolecular gels exhibiting the before mentioned properties were reported, among others [17], pH and acid-responsive sterol-amino acid conjugates [35,36] and bile acid derivatives [37].

In a conventional gel preparation, gelators are synthesized beforehand and then dissolved in suitable solvents. However, in some cases gelators can be synthesized by mixing two or more components directly in a gelling solvent, where new covalent bonds between the building blocks are formed. This modern approach is called *in situ* gelation and recently some gelators of this type were reported [38,39,40]. It was shown that in comparison to the classic method, the *in situ* gelation often: (i) omits heating of the sample; (ii) occurs at ambient temperature; (iii) requires shorter gelation time; or (iv) takes place also in solvents which are not gelled by the conventional gelation [39]. Moreover, the components are needed in a certain ratio to achieve an effective gelator formation, therefore, a lack or excess of one of the components can be used as a tool to control the gelation process.

In this work we present a synthesis of cholesteryl gelator containing an imine bond. Cholesterol and its derivatives are commonly used in the preparation of LMWGs due to the hydrophobic character of the cholesteryl unit and its strong tendency to self-aggregate. Many cholesterol-based gelators have been reported [17,18,19] and they have been often classified as ALS, A(LS)_2_, LS, and LS_2_ types according to the numbers of aromatic (A) and steroidal (S) units, and linkers (L) [18].

## 2. Results and Discussion

### 2.1. Preparation and Gelation Behaviour of Imine ***3***

**Scheme 1:**
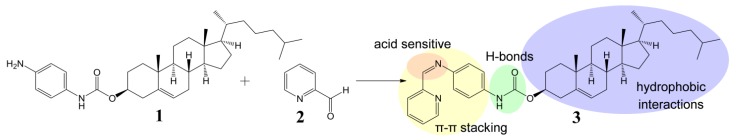
Design and synthesis of imine **3**—gelator of an ALS type [18].

The cholesteryl moiety **1** and 2-pyridinecarboxaldehyde (**2**) were used to prepare gelator **3** of an ALS type (Scheme 1). In our design *p*-phenylenediamine was chosen as a linker in order to synthesize stable (conjugated) imine **3**, and to introduce an amide moiety (*i.e.*, an acceptor and donor for intermolecular H-bonds) to the structure. The imine bond was embedded into the gelator structure due to: (i) its known sensitivity towards acids which we wanted to exploit as an acid-sensitive system; and (ii) its relatively rapid and easy formation at mild conditions, which allows an *in situ* gelator formation. To test these hypotheses, a formation and an acid catalysed hydrolysis of imine **3** were carried out and monitored by NMR. The reaction of amine **1** with the aldehyde **2** occurred at room temperature and it was completed within few hours providing time-stable imine **3** (ESI, Figure S1). However, when the imine **3** was treated with a catalytic amount of *p*-toluenesulphonic acid, it decomposed into its two precursors (protonated compound **1**, and compound **2**) within several minutes (the majority of **3** decomposed within 45 min, see ESI, Figure S2 for details), confirming that the cleavage of the imine linkage under acidic conditions is facile and effective.

**Figure 1:**
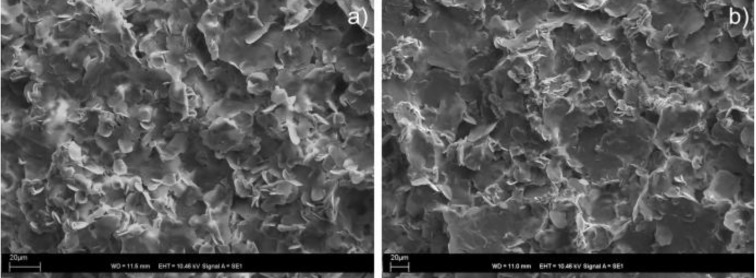
SEM micrographs of xerogels from propan-1-ol of (**a**) of the *in situ* prepared imine **3**; and (**b**) the synthetically prepared imine **3**.

The gelation behaviour of individual compounds was studied in 16 different organic solvents and in water. The tests were carried out for both moieties separately (*i.e.*, for **1**, and for **2**), and for the synthetically and the *in situ* prepared imine **3** (Table 1). Expectably, 2-pyridinecarboxyaldehyde (**2**) did not gelate any of the tested solvents at the concentration of 2% w/v, and amine **1** formed gels only in cyclohexane and DMSO. However, after mixing these two components together and the heating/cooling cycle, gel formation was observed in DMF and DMSO, and in higher alcohols starting from propan-1-ol. The same results were obtained when the gelation behaviour of a synthetically prepared imine **3** was tested. The corresponding imine gels (formed by synthetically and *in situ* prepared gelators) exhibited the same visual properties and the morphology of their xerogels, studied by SEM, was also very similar. The SEM micrograph did not reveal any regular fibrous structures. It only showed three-dimensional structures constructed from thin microflakes of different sizes and mostly rounded shapes (Figure 1, and ESI, Figure S11) suggesting that the gel microstructure was very fragile and collapsed upon solvent evaporation. We also tested the effect of the component ratio on the *in situ* gelation (ESI, Tables S2 and S3). As expected, we found that the effective gelation was reached when a 1:1 molar ratio of the components was used. An excess of 2-pyridinecarboxaldehyde did not significantly affect the gelation process, whereas the excess of amine **1** resulted in a sample precipitation (ESI, Table S2). We also investigated a possibility to prepare gel *in situ* omitting the heating/cooling cycle, which was recently reported [39]. Unfortunately, we did not observe an effective gelation at room temperature, not even after 24 h or ultrasonic treatment, suggesting that the reaction does not quantitatively occur in alcohols at room temperature and energy needs to be added to the reaction via heating.

**Table 1:** Gelation tests (2% w/v).

Solvents	1	2	3 (“ *in situ*”)	3	Solvents	1	2	3 (“ *in situ*”)	3
toluene	S	S	P	P	hexan-1-ol	S	S	**pG**	**pG**
CH_2_Cl_2_	S	S	P	P	heptan-1-ol	S	S	**pG**	**pG**
CHCl_3_	S	S	S	S	octan-1-ol	S	S	**pG**	**pG**
CCl_4_	S	S	P	P	water	I	S	**-**	I
methanol	P	S	I	I	DMF	S	S	**pG**	**pG**
ethanol	P	S	I	I	DMSO	**G**	S	**G**	**G**
propan-1-ol	S	S	**G**	**G**	cyclohexane	**G**	I	-	I
butan-1-ol	S	S	**G**	**G**	hexane	I	I	-	I
pentan-1-ol	S	S	**G**	**G**					

*Note*: S = soluble, P = precipitate upon cooling, G = gel, pG = partial gel, I = insoluble at the solvent boiling point.

**Figure 2:**
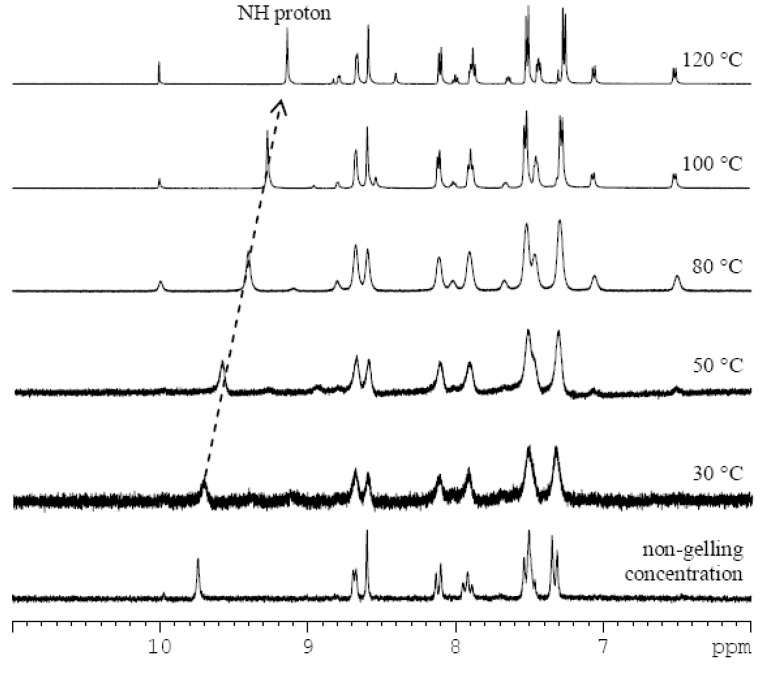
^1^H-NMR subspectra of imine **3** in DMSO-*d_6_* at non-gelling concentration (0.2% w/v), and as a DMSO-*d_6_* gel (2.5% w/v) at different temperatures (30–120 °C) with a highlighted shift of the amide proton signal (dotted arrow).

In order to discover the non-covalent interactions involved in the gelation process, we carried out variable-temperature ^1^H-NMR measurements of the imine **3** gel in DMSO-*d_6_* (Figure 2, and ESI, Figures S4 and S5). By heating the sample in 10 °C steps (30–120 °C), the broad signals gradually turned sharper. The well-resolved spectral patterns were finally observed at 100 °C, suggesting that the gel fully transformed to the solution state. As a control experiment, a ^1^H-NMR spectrum of imine **3** in a non-gelling concentration (0.2% w/v) was recorded (Figure 2). Chemical shifts of proton signals of imine **3** at the non-gelling concentration match those in the gel state at 30 °C indicating that the observed peaks correspond to the free molecules which are not tightly integrated in the gel network. With an increase of temperature, the chemical shifts of the signals did not change remarkably with the sole exception of the amide proton. The chemical shift values of N-H changed from 9.71 ppm at 30 °C to 9.13 ppm at 120 °C. This indicates an increase in the strength of the N-H bond, direct evidence of the disentanglement of NH from a bonding state. This disruption of intermolecular hydrogen-bonding can be also indirectly observed by a small deshielding of signals of the protons of the phenyl ring (the strongest change is from 7.33 at 30 °C to 7.27 ppm at 120 °C).The experiment did not prove the presence of π-π stacking between pyridine units in the DMSO-gel, because only small shielding (0.01–0.05 ppm) of pyridine protons was observed. The spectra also showed that the increase of temperature in the presence of water (from the deuterated solvent) caused partial hydrolysis of imine **3** to the starting components **1** and **2**.

The presence of intermolecular hydrogen bonds was further confirmed by a dilution NMR experiment. The ^1^H-NMR spectra were recorded for the imine **3** at different concentration and showed deshielding of the NH signal at higher concentrations indicating the intermolecular hydrogen bond formation (ESI, Figure S3). Overall, we propose that the gel formation is caused by an aggregation of cholesteryl units *via* hydrophobic and van der Waals interactions in polar solvents, and that the structure is further stabilized by intermolecular hydrogen bonds between amide groups and possibly by π-π interactions between the aromatic parts of the molecules.

### 2.2. Acid-Responsive Gels

**Scheme 2:**
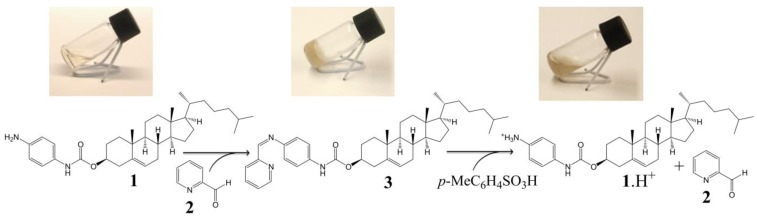
*In situ* gelation and acid-induced gel-sol transition of **3** in propan-1-ol.

Considering the difference in the gelation behaviour of amine **1** and imine **3**, and after successful acid catalysed hydrolysis of imine **3** monitored by NMR (ESI, Figure S2), we expected that the addition of a small amount of acid to the gel of imine **3** should result in a gel-sol transition. When we placed a catalytic amount of *p*-toluenesulphonic acid on the top of a gel of imine **3** in propan-1-ol, butan-1-ol, pentan-1-ol, and DMSO, a gel-sol transition occurred (Scheme 2, and ESI, Figure S10). We suppose that the hydrolysis of the imine linkage weakened the intermolecular interactions, resulting in the dissociation of the self-assembled network of the gelators and giving rise to an acid-mediated gel-sol transition. This process occurred within less than 2 h, and could be speeded up to several minutes under an ultrasonic treatment. In another experiment, a drop of hydrochloric acid (1 M aqueous solution) was added resulting in a gel-sol transition within several minutes (approx. 15 min). And again, the process could be speeded up to few minutes by an ultrasonic treatment. These results, together with a recently published report [41], show that the organogels formed by imines are acid-responsive. We believe that such systems could find potential applications in drug release systems and therefore, gels based on imine **3** were further studied.

### 2.3. Two Component Gel System

**Figure 3:**
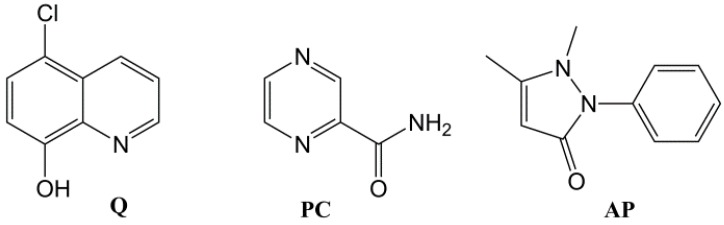
Chemical structures of the chosen drugs: 5-chloro-8-hydroxyquinoline (**Q**), pyrazinecarboxamide (**PC**), and antipyrine (**AP**).

In order to study the potential of gelator **3** in drug delivery [12,13,14], two component gels [42,43] containing different drugs were prepared. As a second component, aromatic drugs of different polarities were chosen: 5-chloro-8-hydroxyquinoline (cloxyquine, **Q**), pyrazinecarboxamide (pyrazinamide, **PC**), and antipyrine (phenazone, **AP**) (Figure 3). The quinoline derivative and pyrazinecarboxamide are drugs used in the treatment of tuberculosis [44,45], and antipyrine is analgesic and antipyretic. These pharmaceuticals were chosen because of their aromatic character (*i.e.*, possibility to intercalate to the aromatic parts of imine **3**), commercial availability, low price, and gelation potential (recently, 5-chloro-8-hydroxyquinoline was found to be an effective gelator for alcohol-water mixtures [46]).

**Figure 4:**
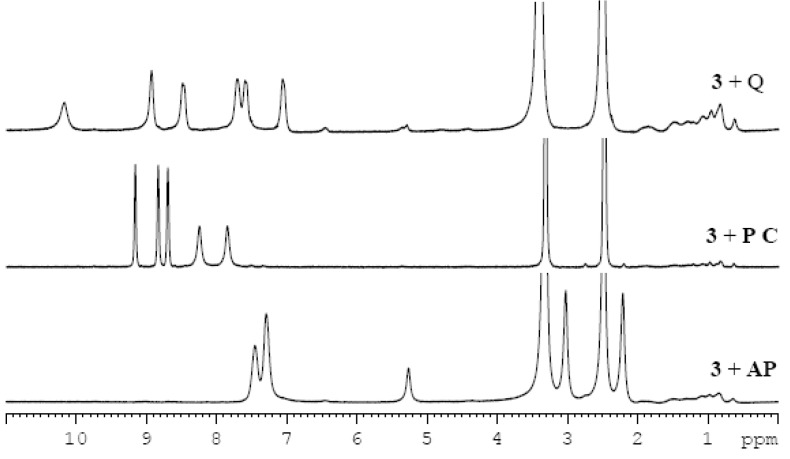
Comparision of the ^1^H-NMR spectra of the different imine-drug gels in DMSO-d*_6_* (3% w/v).

It was found that the mixtures of imine **3** and the drugs in a 1:1 ratio formed gels in most of the selected solvents (Table 2). In order to investigate interactions between the gelator and drug molecules, ^1^H-NMR spectra of imine-drug gels (Figure 4) and variable temperature ^1^H-NMR spectra of a gel of imine **3** and pyrazinecarboxamide were recorded (ESI, Figures S6 and S7). The drug signals in the spectra are not sharp suggesting that the mobility of the drug molecules is restricted within the gel network. As it was proposed by Miravet *et al.* [47], broadening of the signals, indicating shorter *T_2_* relaxation times, is often caused by a fast exchange between molecules in liquid-like (“visible” by NMR) and solid-like (“invisible” by NMR) phases, which means in our case between the free dissolved drug molecules and the drug molecules interfering in the gelator network. With an increase of temperature the gel melts and the drug is fully released to the solution and as a consequence, the signals become sharper. The fully sharp signals were observed at 100 °C (see Figures S6 and S7) which corresponds to the gel-sol transition of the gel of imine **3** (Figure 1). Unfortunately, the experiment did not prove the presence of π-π stacking either between drug molecules or between drug and gelator molecules in a DMSO-gel, because additional shifts, besides the small upfield shifts (approximately 0.05 ppm) of the aromatic proton signals expected for an increase of temperature, are missing.

**Table 2:** Gelation tests (2% w/v).

Solvents	3	Q	3 + Q	PC	3 + PC	AP	3 + AP
ethanol	I	R		P	I	S	I
propan-1-ol	**G**	**G**	**G**	P	**G**	S	**G**
butan-1-ol	**G**	R	**G**	P	**G**	S	**G**
pentan-1-ol	**G**	R	**pG**	P	**G**	S	**G**
hexan-1-ol	**pG**	R	**pG**	P	**G**	S	**G**
octan-1-ol	**pG**	R	**pG**	P	**pG**	S	**pG**
DMF	**pG**	S	**pG**	S	**pG**	S	**pG**
DMSO	**G**	S	**G**	S	**G**	S	**G**

*Note*: S = soluble, P = precipitate upon cooling, G = gel, pG = partial gel, R = recrystallization upon cooling, I = insoluble at the solvent boiling point.

**Figure 5:**
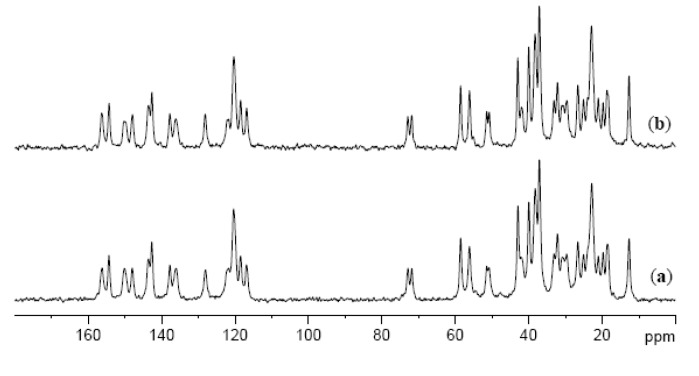
^13^C CPMAS NMR spectra of (**a**) xerogel of **3** from propan-1-ol; (**b**) xerogel of **3** and **PC** (1:1) from propan-1-ol.

In order to investigate an influence of a drug on the molecular packing of the gelator, ^13^C cross polarization magic angle spinning (CPMAS) NMR spectra of xerogels of imine **3**, and of imine **3** and pyrazinecarboxamide in a 1:1 ratio were recorded. As can be seen from Figure 5 (and Figure S8 in ESI), the spectra display very similar patterns suggesting that the presence of a drug does not significantly affect the packing mode of imine **3** in the xerogel state. Moreover, the spectra show that imine **3** is much more crystalline compared to the drug because the drug signals can be hardly seen in the spectrum. Some of the signals reveal a double resonance pattern indicating the samples being either (i) a mixture of different polymorphic forms, or (ii) composed of a form having two non-equivalent molecules present in an asymmetric unit. A similar observation has been reported earlier for bile acid-based gelators and it was shown to be due to the presence of two non-equivalent molecules in the crystal lattice [48].

The morphology of the xerogels of the imine-drug gels from pentan-1-ol and DMSO was studied by SEM (ESI, Figure S11). Micrographs of xerogels of imine **3** from alcohols and from DMSO displayed similar microstructures regardless of the presence or absence of the drugs suggesting that the drug molecules did not significantly affect the way of self-assembly of the gelator. Only images of the xerogel of **3+PC** from pentan-1-ol showed a slightly different arrangement with partly integrated elongated crystals (ESI, Figure S11b). These crystals could represent drug molecules which were not too strongly incorporated to the gel network and could freely crystallize during the sample preparation when the solvent was slowly evaporated.

### 2.4. Controlled Drug Release

**Figure 6:**
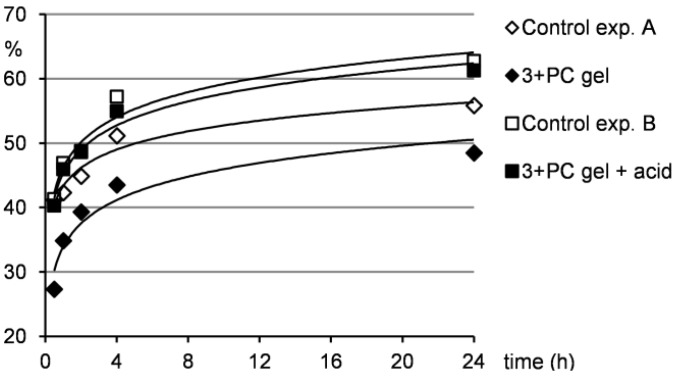
Release of *PC* from the gel to the water layer under neutral and acidic conditions.

**Figure 7:**
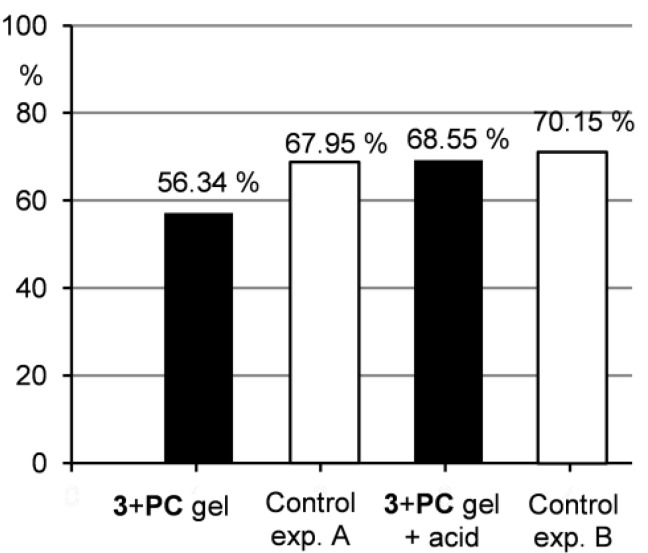
Percentage of PC released to the water layer after ultrasonic treatment.

In order to examine the potential of the two component gels in drug delivery [12,13,14], the release of a drug from gel to water was studied. For this purpose, we chose a gel of imine **3** and pyrazinecarboxamide in pentan-1-ol. Pyrazinecarboxamide was selected as a model drug due to its suitable solubility in water (15 g/L at 25 °C). Gels in pentan-1-ol were chosen because of their stability and because pentan-1-ol forms a two layered system with water, which made the drug release experiments easy to monitor. Gel samples of imine **3** and pyrazinecarboxamide were treated with water either without or with *p*-toluenesulfonic acid. After 0.5, 1, 2, 4 and 24 h, the water layers were separated off and the amount of the released drug was analysed by NMR measurements. Control experiments, in which pyrazinecarboxamide was dissolved in pentan-1-ol and treated either with water (exp. A) or with acidic water (exp. B), were carried out as well. To check the drug release under non-calm conditions, the samples were treated by ultrasonic for 10 min and after one hour of standing without any additional disturbance, the water layers were checked (in the same way like in the other drug release experiments). The results are summarized in Figure 6 and Figure 7 (for details see ESI, Figure S13 and Table S4).

Due to the fact that water is more dense than the gel from pentan-1-ol, the water put above the gel sample diffused through the gel and thus lifted the gel layer to the top (within the first 2–4 h). Naturally, this water penetration through the gel washed out the major amount of the drug in the first several hours of the experiment as can be seen from Figure 6.

Furthermore, Figure 6 shows that the drug release from the gel samples was smaller compared to the control experiment A, but still occurred. This indicates that the drug is not strongly integrated to the gel network and can be washed out when water penetrates the gel as described above. Therefore, such system could find potential applications in a slow drug release, where a distribution in small quantities over longer time is needed. Contrarily, when the gel samples were treated with acidic water, the situation changed significantly. Under acidic conditions, a gel-sol transition occurred and as a consequence, the drug release to the water layer was much quicker compared to the situation when the gel was treated only with water. The results of the drug release from the gel under acidic conditions were comparable to those of the control experiment B indicating that the gel hydrolysis and subsequent drug release were efficient. This outcome supports the idea that acid-responsive gel systems with entrapped pharmaceuticals could be effectively used in drug delivery when pH-induced drug release is needed.

## 3. Experimental

### 3.1. General

Analytical grade reagents and solvents were used for the synthesis, purification and gelation studies. Cholesteryl chloroformate was purchased from Alfa Aesar (Karlsruhe, Germany), and *p*-phenylenediamine and 2-pyridinecarboxaldehyde from Sigma Aldrich (Steinheim, Germany). 2-pyridinecarboxaldehyde was freshly distilled before use. Pharmaceuticals for the drug release experiments were purchased from Sigma Aldrich (pyrazinecarboxamide and 5-chloro-8-hydroxyquinoline) and from Fluka (Steinheim, Germany) (antipyrine). ^1^H and ^13^C-NMR experiments were run with a Bruker Avance DRX 500 NMR spectrometer equipped with a direct observation BBO probe head working at 500.13 MHz for ^1^H and at 125.76 MHz for ^13^C-NMR spectra were measured in CDCl_3_ and the chemical shifts were referenced to the solvent signal (δ = 7.26 ppm for ^1^H, and δ = 77.0 ppm for ^13^C). The numbering of the steroidal part is according to the IUPAC rules. Molecular masses were measured either by using a Micromass LCT ESI-TOF mass spectrometer or by a VG AutoSpace 3500 HR-MS high resolution mass spectrometer. IR spectra were recorded on a Bruker Tensor 27 FTIR spectrometer.

### 3.2. Preparation of compound ***1***

compound **1** was prepared according a previously reported procedure [49]. The desired product was obtained as a light yellow solid in 34% yield. δ_H_ (500.13 MHz; CDCl_3_): 0.68 (3H, s, 18-CH_3_), 0.87 (6H, dd, *J* = 2.2; 6.6 Hz, 26-CH_3_ + 27-CH_3_), 0.92 (3H, d, *J* = 6.5 Hz, 21-CH_3_), 1.03 (3H, s, 19-CH_3_), 4.58 (1H, m, 3-CH), 5.39 (1H, m, 6-CH), 6.33 (1H, bs, NH), 6.64 (2H, benzene ring), 7.14 (2H, benzene ring). δ_C_ (125.7 MHz; CDCl_3_): 11.86 (C-18), 18.72 (C-21), 19.33 (C-19), 21.06 (C-11), 22.55 (C-27), 22.80 (C-26), 23.84 (C-23), 24.29 (C-15), 28.00 (C-25), 28.14 (C-2), 28.22 (C-16), 31.90 (C-7), 31.91 (C-8), 35.79 (C-20), 36.20 (C-22), 36.59 (C-10), 37.01 (C-1), 38.52 (C-4), 39.52 (C-24), 39.76 (C-12), 42.33 (C-13), 50.04 (C-9), 56.17 (C-17), 56.71 (C-14), 74.66 (C-3), 115.62 (benzene ring), 121.00 (benzene ring), 122.61 (C-6), 129.46 (benzene ring), 139.74 (C-5), 142.59 (benzene ring), 153.58 (C=O); ν_max_/cm^−1^: 3407, 3332 (NH), 2936, 2866 (CH), 1728 (C=O, -O), 1631 (C=O, -NH), 1525 (NH, bending) and 1208 (C-O); *m/z* (ES^+^) 543.43 ([M+Na]^+^, 100%), 1063.85 ([2M+Na]^+^, 27%); *m/z* (HR-ESI) 521.4101, [C_34_H_52_N_2_O_2_+H]^+^ requires 521.4102; 543.3933, [C_34_H_52_N_2_O_2_+Na]^+^ requires 543.3921.

### 3.3. Preparation of Compound ***3***

compound **1** (200 mg, 0.384 mmol) was dissolved in dry CH_2_Cl_2_ (8 mL), then freshly destilled 2-pyridinecarboxyaldehyde (**2**) was added (36.5 µL, 0.384 mmol). The mixture was stirred at rt under N_2_ atmosphere for 18 h. After a solvent evaporation, product 3 was obtained as a light orange solid in a quantitative yield (233 mg). δ_H_ (500.13 MHz; CDCl_3_): 0.68 (3H, s, 18-CH_3_), 0.87 (6H, dd, *J* = 2.2; 6.6 Hz, 26-CH_3_ + 27-CH_3_), 0.92 (3H, d, *J* = 6.5 Hz, 21-CH_3_), 1.03 (3H, s, 19-CH_3_), 4.62 (1H, m, 3-CH), 5.40 (1H, m, 6-CH), 6.73 (1H, bs, NH), 7.30 (2H, benzene ring), 7.34 (1H, pyridine ring), 7.44 (2H, benzene ring), 7.79 (1H, pyridine ring), 8.18 (1H, pyridine ring), 8.62 (1H, s, imine-H), 8.70 (1H, pyridine ring). δ_C_ (125.7 MHz; CDCl_3_): 11.85 (C-18), 18.71 (C-21), 19.31 (C-19), 21.05 (C-11), 22.54 (C-27), 22.79 (C-26), 23.84 (C-23), 24.27 (C-15), 27.99 (C-25), 28.09 (C-2), 28.21 (C-16), 31.87 (C-7), 31.90 (C-8), 35.78 (C-20), 36.19 (C-19), 36.58 (C-10), 36.97 (C-1), 38.45 (C-4), 39.51 (C-24), 39.74 (C-12), 42.32 (C-13), 50.02 (C-9), 56.16 (C-17), 56.69 (C-14), 75.08 (C-3), 119.27 (benzene ring), 121.74 (pyridine ring), 122.10 (benzene ring), 122.77 (C-6), 124.91 (pyridine ring), 136.58 (pyridine ring), 137.15 (benzene ring), 139.57 (C-5), 145.97 (benzene ring), 149.66 (pyridine ring), 153.00 (C=O), 154.76 (pyridine ring), 159.21 (C=N); ν_max_/cm^−1^: 3407, 3332 (NH), 2934, 2866 (CH), 1727 (C=O, -O), 1626 (C=O, -NH), 1524 (NH, bending) and 1225 (C-O); *m/z* (ES^+^) 610.46 ([M+H]^+^, 84%), 632.46 ([M+Na]^+^, 100%), 1241.94 ([2M+Na]^+^, 25%); *m/z* (HR-ESI) 610.4363, [C_40_H_55_N_3_O_2_+H]^+^ requires 610.4367.

### 3.4. Gelation Tests

In a typical gelation test a weighed amount of the gelator was mixed with a measured volume of the selected solvent in a sealed 5 mL test tube. The sample was sonicated for *ca*. 2–3 min and then the mixture was heated until the solid was completely dissolved (if soluble). The resulting solution was allowed to slowly cool down to room temperature. Finally the test tubes were inverted to observe if the contents could still flow. Upon cooling down, the formation of gel (G), precipitate (P), or solution (S) was detected.

### 3.5. NMR Studies

^1^H-NMR spectra of imine **3** formation and hydrolysis, ^1^H-NMR spectra of imine-drug gels and ^1^H-NMR spectra for the drug release experiments were recorded with a Bruker Avance DPX 250 spectrometer equipped with a 5mm 1H/BB inverse detection probe head working at 250.13 MHz for ^1^H. Variable-temperature ^1^H-NMR spectra of gels were recorded with a Bruker Avance DRX 500 NMR spectrometer equipped with a BBO probe head working at 500.13 MHz for ^1^H. The gel samples were prepared directly in an NMR tube; a weighed amount of a gelator, or a mixture of a gelator and drug, was dissolved upon heating in 0.6 mL of DMSO-*d_6_*, and the gel samples were stabilized overnight. The VT ^1^H-NMR experiments were conducted varying the temperature by 10 °C steps. The samples were allowed to stabilize for 5 min at each temperature before acquiring the spectra.

^13^C CPMAS NMR spectra were recorded with a Bruker AV 400 spectrometer equipped with a 4 mm standard bore CPMAS probe. The dried and finely powdered samples were packed in ZrO_2_ rotors. The experiments were carried out at a 10 kHz spinning rate under Hartman-Hahn condition at the contact time being 2 ms with 5 s recycle delay. The number of scans varied between 400–1,000. FIDs were zero filled twice and apodized by 20 Hz exponential window function prioir to Fourier transform (FT). The ^13^C chemical shift was calibrated using a carbonyl signal of a glycine sample at 176.03 ppm as an external standard. Complete lists of acquisition and processing parameters are available by E. K. on request.

### 3.6. SEM Measurments

Scanning electron micrographs of xerogels were taken on a Bruker Quantax400 EDS microscope equipped with a digital camera. The samples of the xerogels were prepared by placing a hot, clear solution of the gelator on carbon tape over a sample stub. The samples were dried at room temperature and then sputter coated with a thin layer of gold in a JEOL Fine Coat Ion Sputter JFC-1100.

### 3.7. Drug Release Experiments

The gels of imine **3** and pyrazinecarboxamide (2.8% w/v), prepared in a 1:1 ratio in 0.5 mL of pentan-1-ol (n_PC_ = 0.0195 mmol), were stabilised overnight. Then water (0.5 mL) either without or with *p*-toluenesulfonic acid (0.0053 mmol) was added. The samples stayed without any shaking or other type of disturbance. Water layers (0.4 mL) were separated off at certain times (after 0.5, 1, 2, 4 and 24 h), and after solvent evaporation, they were dissolved in 0.6 mL of D_2_O and analysed by NMR with succinic acid (0.0042 mmol) as an internal standard. As control experiments (A and B), pyrazinecarboxamide (0.0195 mmol) was dissolved in pentan-1-ol (0.5 mL), and the samples were treated in the same way as the gel samples (adding of 0.5 mL of water either without or with 0.0053 mmol of *p*-toluenesulfonic acid, and then analysed by NMR with 0.0085 mmol of succinic acid as an internal standard). The percentage of the released drug was calculated from the peak area of drug signals of a sample to the peak area of drug signals of a reference sample which was prepared by dissolving pyrazinecarboxamide (0.0195 mmol) in 0.6 mL of D_2_O with succinic acid (0.0085 mmol) as an internal standard.

## 4. Conclusions

In summary, imine **3** represents the first gelator which forms *in situ* acid-responsive supramolecular gels. The gelator can also be prepared synthetically beforehand. In both cases the resulting gels exhibit the same macroscopic and microscopic properties without any loss by the *in situ* generation of the gel. The modern *in situ* approach to gel formation is still very rare [38,39,40] but should soon see a tremendous increase in popularity considering the advantages already described for these systems [39]. Besides the shortened gel preparation time integral to all *in situ* formations, the system here described can be controlled by changes in the molar ratio of the components. Another speciality of the described gelator is the imide moiety designed into the structure not just to facilitate the *in situ* formation but also to afford an acid-induced hydrolysis. This acid sensitivity we were able to exploit as a drug release system for different aromatic drugs. The drugs were before embedded into the gel network. In contact with water these two-component gels are slowly releasing the drugs constituting a slow drug release system. Contrarily, by adding acid, the drugs can be instantly released from the gel network. We believe that acid-responsive gels containing imine bonds are promising soft materials with many potential applications in drug delivery and controlled release systems [12,13,14].

## Supplementary Materials

Electronic Supplementary Information (ESI) can be accessed at: http://www.mdpi.com/1420-3049/18/4/3745/s1.
