# Supplementary material for: In Situ Formation of Steroidal Supramolecular Gels Designed for Drug Release
Source: Molecules. 2013 Mar 25;18(4):3745–59. doi: 10.3390/molecules18043745 (PMC6270054; doi:10.3390/molecules18043745)

Supplementary Materials

NMR Studies

a) ^{1}H-NMR Study of Imine **3** *Formation:*

Experimental details:

Amine **2** (10 mg) in 580 µL of $CDCl_3$ were mixed with a stock solution of 2-pyridinecarboxaldehyde (20 µL, 22 µL of 2-pyridinecarboxaldehyde + 218 µL of $CDCl_3$) in an NMR tube. ^{1}H-NMR spectra were recorded 3, 5, 15, 30, 45, 60, and 120 min after mixing the components.

Table S1. Conversion of imine **3** in $CDCl_3$ over time.

Time after mixing (min)	Peak area (aldehyde proton)	% Aldehyde	Peak area (imine proton)	% Imine
3	0.8068	80.68	0.1759	17.59
5	0.6398	63.98	0.3626	36.26
10	0.4999	49.99	0.4954	49.54
15	0.3643	36.43	0.6348	63.48
20	0.2938	29.38	0.7052	70.52
30	0.2241	22.41	0.7770	77.70
45	0.1754	17.54	0.8245	82.45
60	0.1413	14.13	0.8436	84.36
120	0.1048	10.48	0.8795	87.95

Figure S1. ^{1}H-NMR study of imine **3** formation in $CDCl_3$ over time.

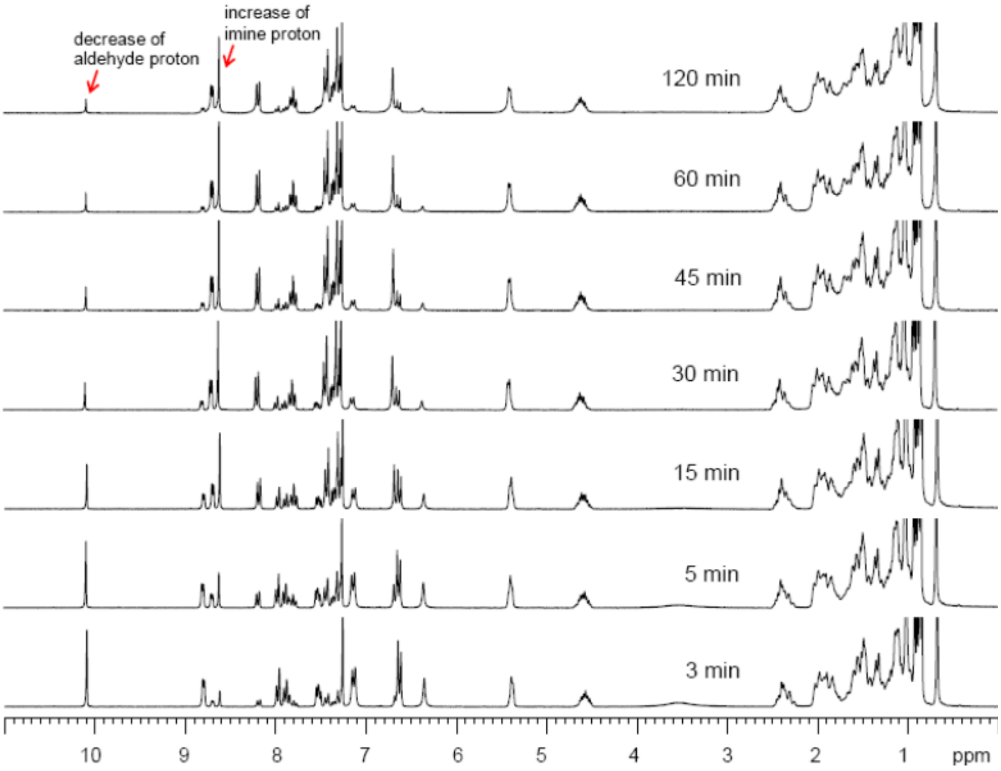

b) ^{1}H-NMR Study of Imine **3** *Hydrolysis:*

Experimental details:

Imine **3** (40.9 mol/mL) was dissolved in $CDCl_3$ (0.6 mL). Then a catalytic amount (<1 mg) of *p*-$MeC_6H_4SO_3H$ was added. ^{1}H-NMR spectra were recorded after 3, 5, 15, 30, 45, and 60 min.

Figure S2. ^{1}H-NMR study of imine **3** hydrolysis by *p*-$MeC_6H_4SO_3H$ in $CDCl_3$ over time.

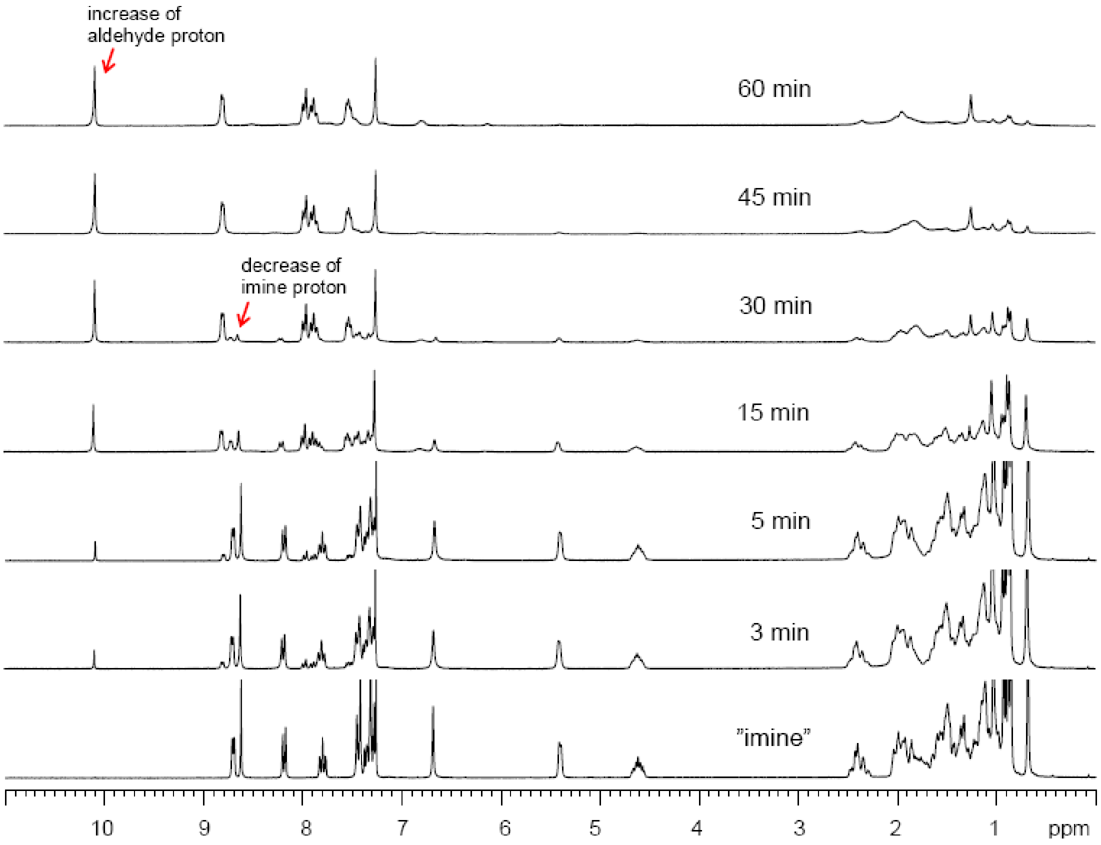

c) ^{1}H-NMR Dilution Study of Imine **3***:*

Figure S3. Aromatic region of the ^{1}H-NMR spectra of imine **3** in $CDCl_3$ at a concentration of (**a**) 1.4×10^{-3} M; (**b**) 2.7×10^{-3} M; (**c**) 5.5×10^{-3} M; (**d**) 1.1×10^{-2} M; (**e**) 2.2×10^{-2} M; (**f**) 2.8×10^{-2} M.

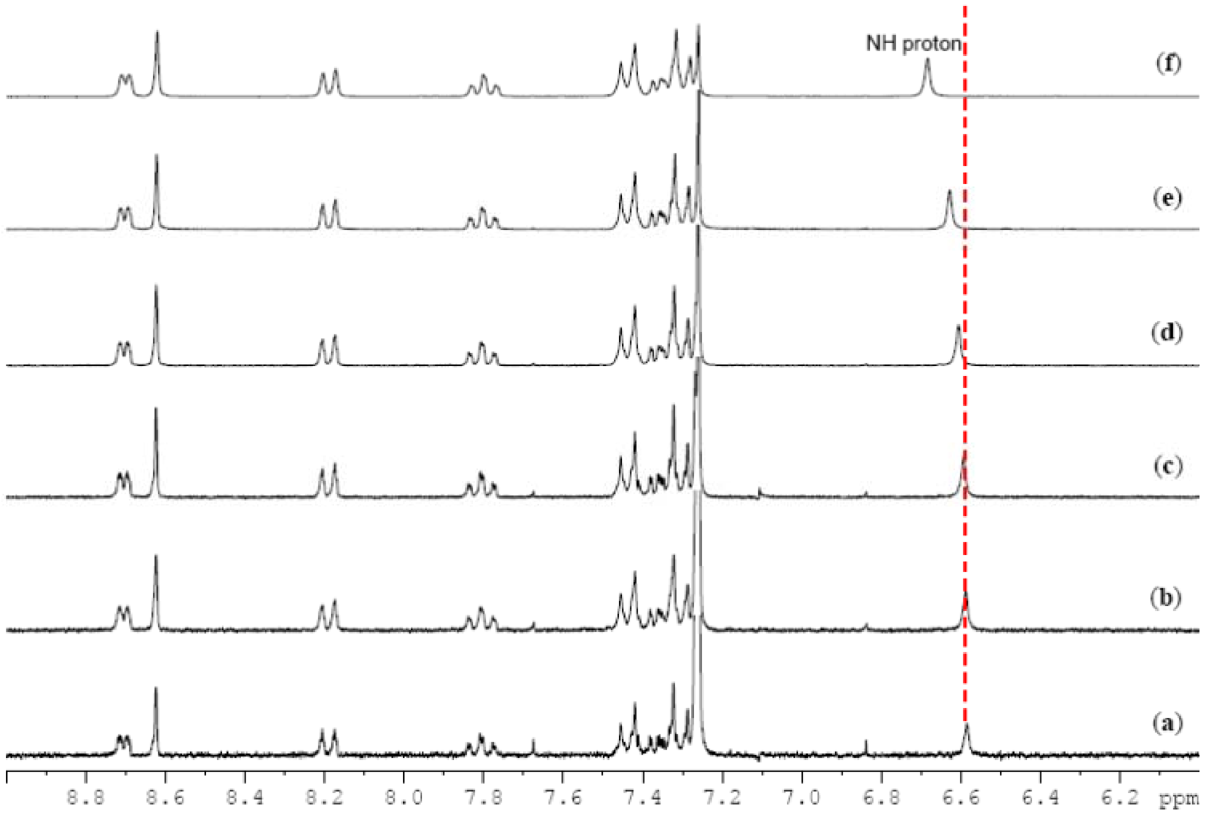

d) VT [1]*H-NMR Spectra of Imine* **3**:

Figure S4. ^{1}H-NMR spectra of imine **3** in DMSO-d_6 at non-gelling concentration (0.2% w/v, bottom spectrum) and as a DMSO-d_6 gel (2.5% w/v) at different temperatures (30–120 °C).

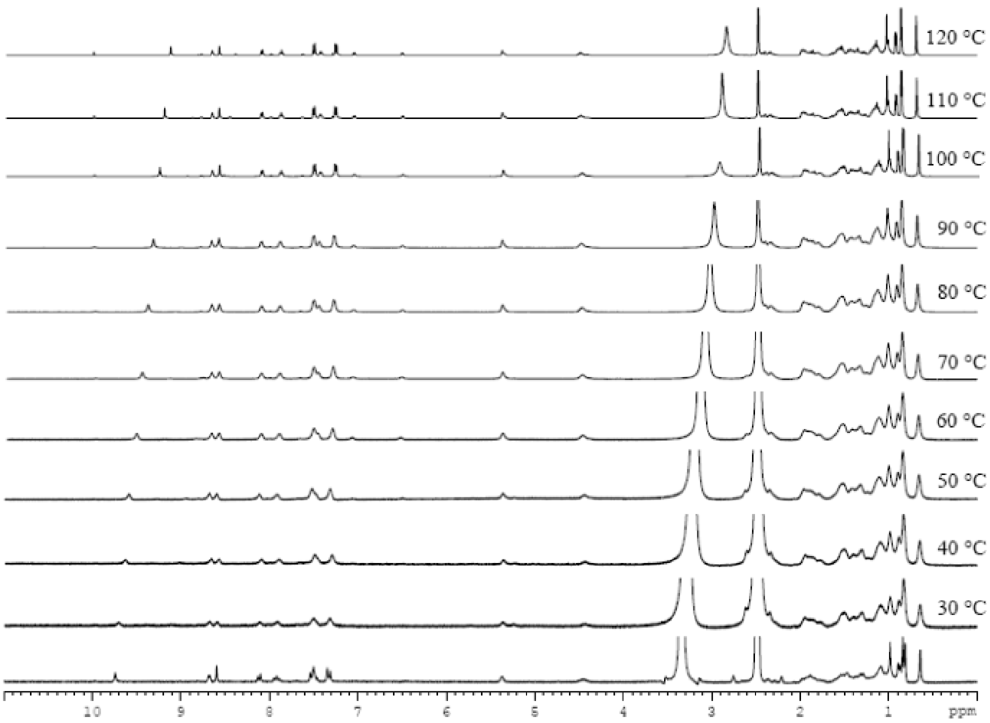

Figure S5. ^{1}H-NMR subspectra of imine **3** in DMSO-d_6 at non-gelling concentration (0.2% w/v, bottom spectrum) and as a DMSO-d_6 gel (2.5% w/v) at different temperatures (30–120 °C).

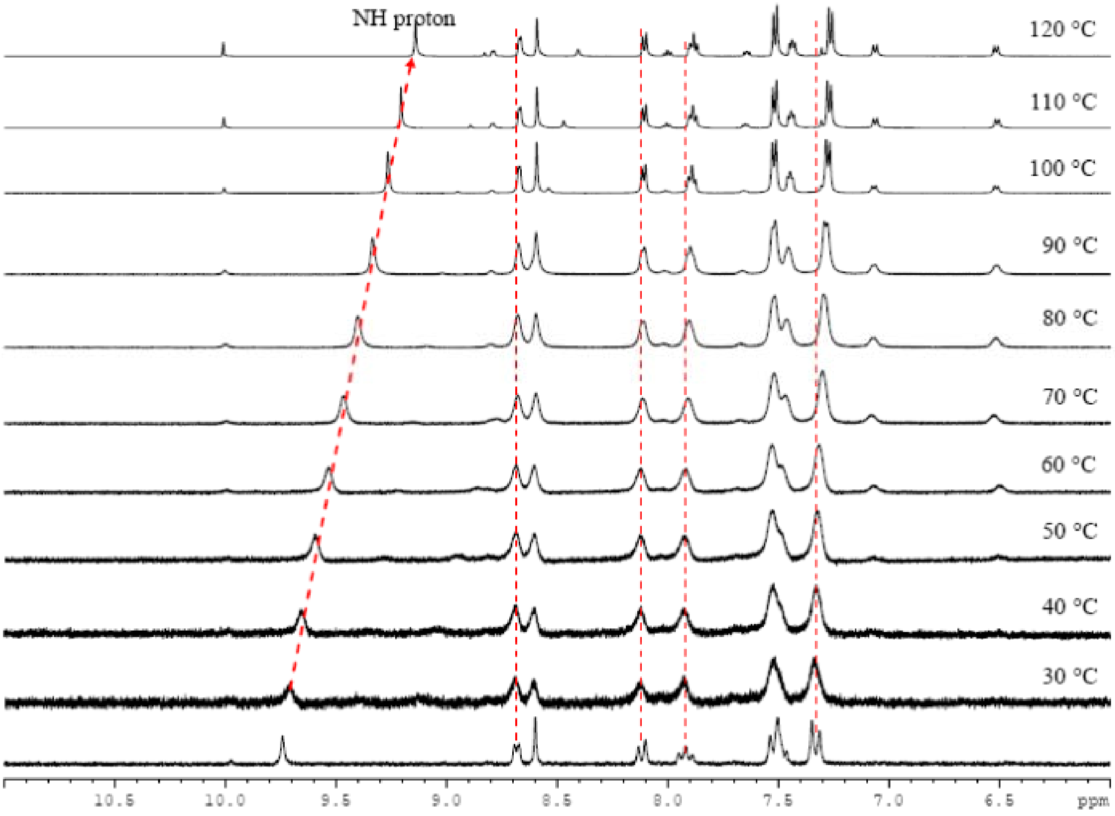

e) VT ^{1}H-NMR Spectra of Imine 3 and Pyrazinecarboxamide:

Figure S6. ^{1}H-NMR spectra of gel of imine **3** and pyrazinecarboxamide (1:1) in DMSO-d_6 (2.8% w/v) at different temperatures (30–120 °C).

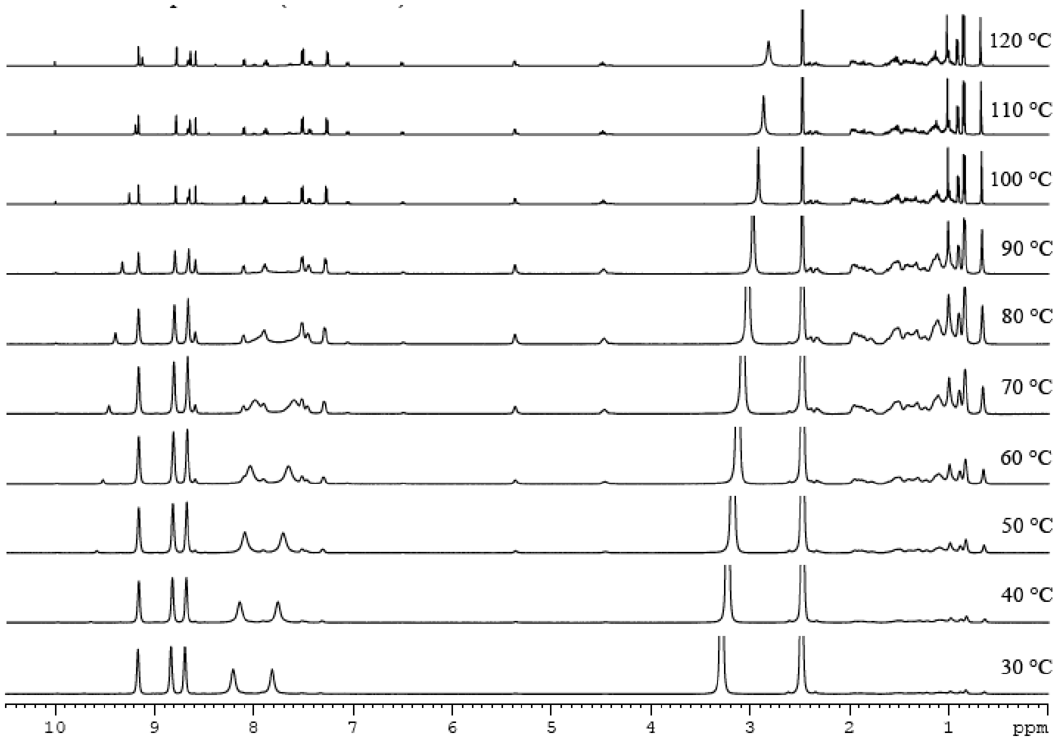

Figure S7. ^{1}H-NMR subspectra of gel of imine **3** and pyrazinecarboxamide (1:1) in DMSO-d_6 (2.8% w/v) at different temperatures (30–120 °C).

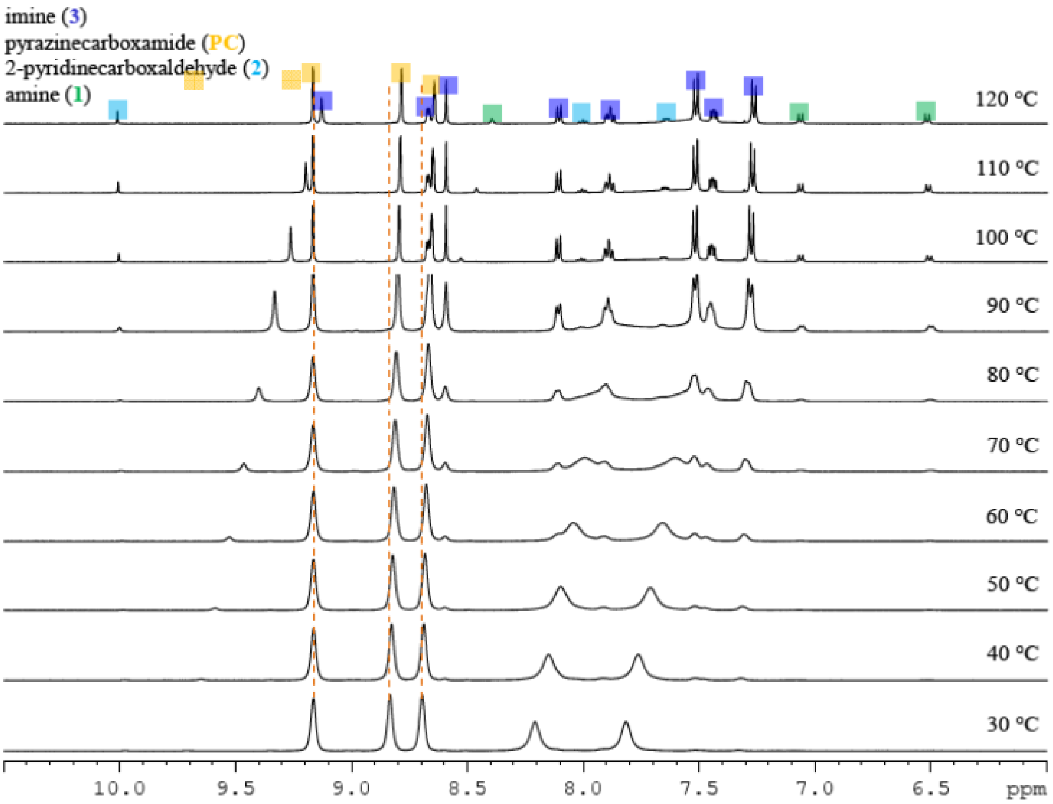

e) ^{13}C CPMAS NMR Spectra:

Figure S8. ^{13}C-NMR spectrum of (**a**) imine **3**, and ^{13}C CPMAS NMR spectra of (**b**) xerogel of imine **3** from propan-1-ol; (**c**) xerogel of imine **3** from pentan-1-ol; (**d**) xerogel of imine **3** and pyrazinecarboxamide (1:1) from propan-1-ol; and (**e**) pyrazinecarboxamide recrystallized from propan-1-ol.

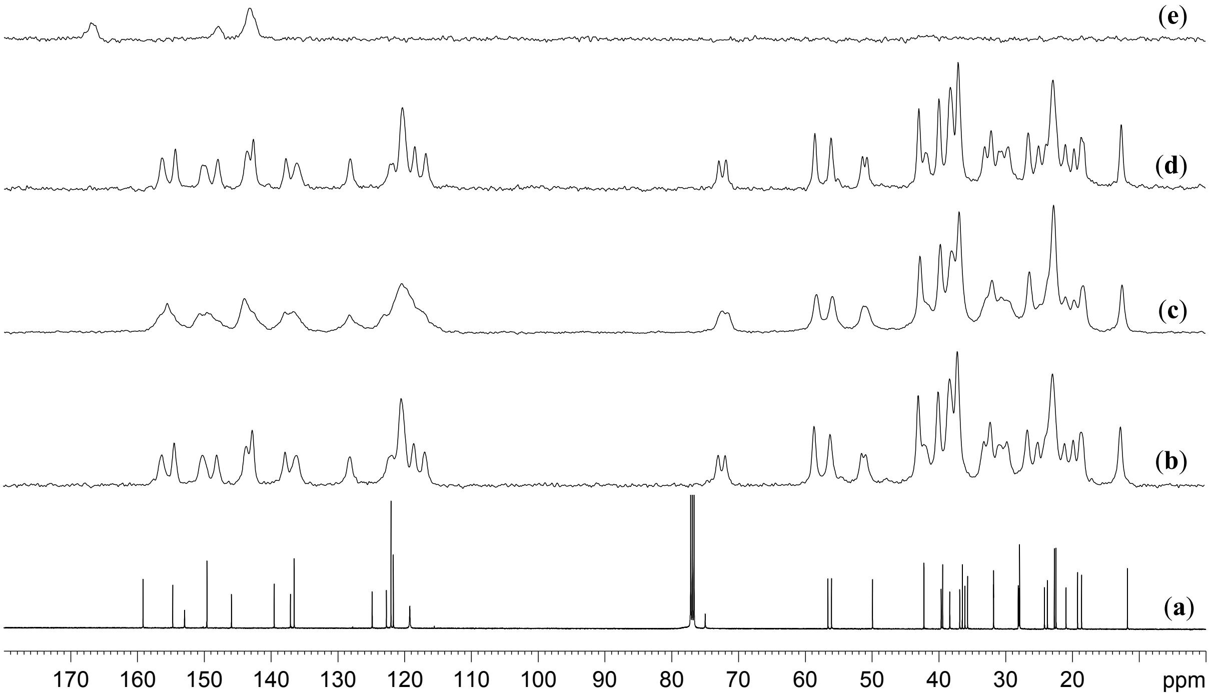

Note: Corresponding signals of the spectra of xerogels closely resemble to each other suggesting that imine **3** behaves very similarly in the xerogel state. The samples of the xerogels from propan-1-ol show relatively sharp signals indicating that they are more crystalline in nature than the sample of the xerogel from pentan-1-ol. Moreover, some signals reveal a double resonance pattern which means that the sample is either (i) a mixture of different polymorphic forms; or (ii) composed of a form having two non-equivalent molecules present in an asymmetric unit.

Photos of Gels

Figure S9. Photographs of gels (2 % w/v) of imine **3** in (**a**) propan-1-ol; (**b**) pentan-1-ol, and (**c**) DMSO; and of imine **3** and pyrazinecarboxamide (1:1) in (**d**) pentan-1-ol; and (**e**) DMSO.

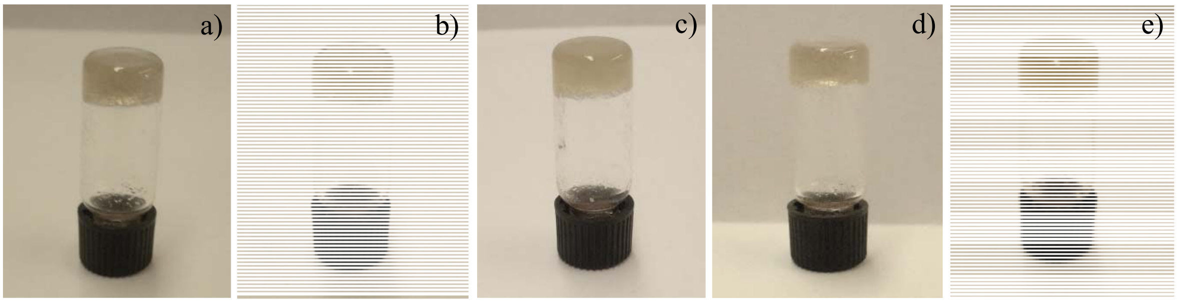

Figure S10. Photographs of gel of imine **3** in propan-1-ol (2% w/v) after addition of 25 µL of 0.1 M aqueous solution of *p*-$MeC_6H_4SO_3H$.

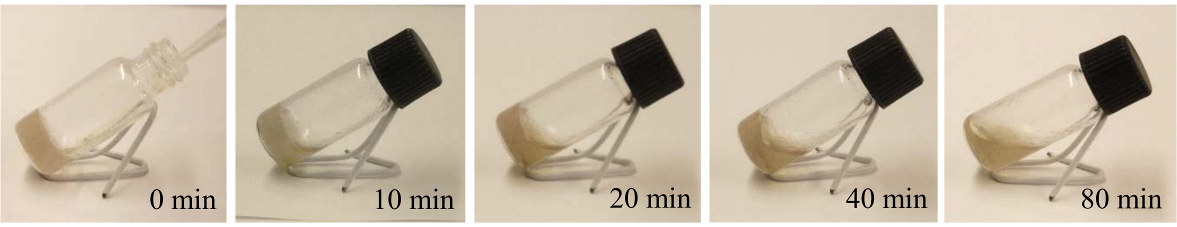

Additional SEM Micrographs

Figure S11. SEM micrographs of xerogels of imine **3** in (**a**) pentan-1-ol and (**d**) DMSO; of imine **3**+**PC** in (**b**) pentan-1-ol and (**e**) DMSO; and of imine **3**+**AP** in (**c**) pentan-1-ol and (**f**) DMSO.

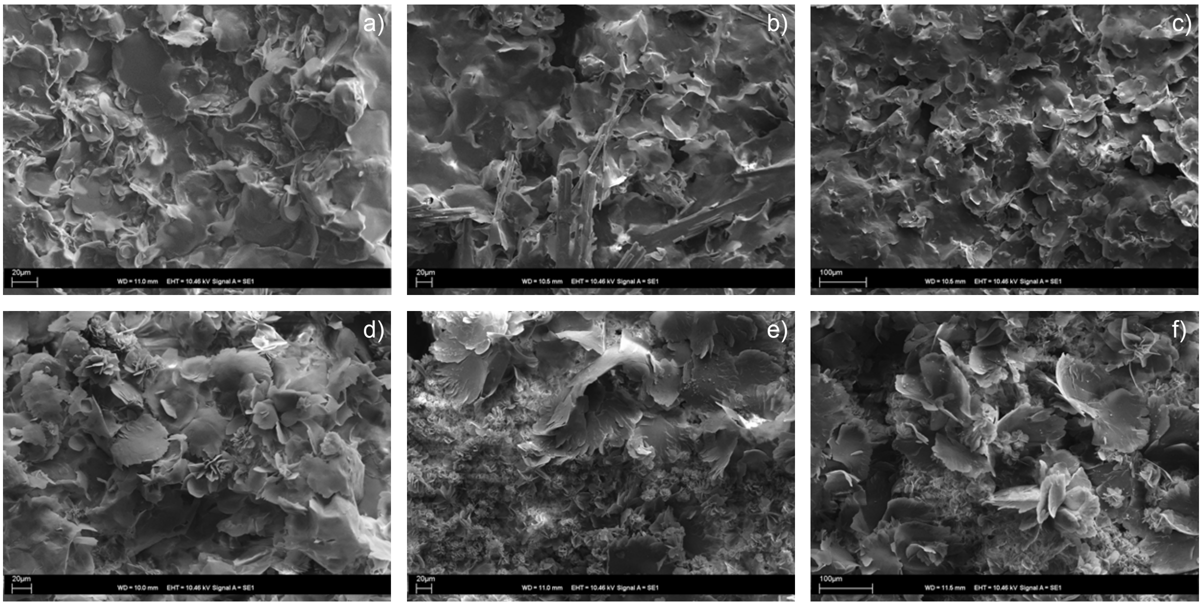

In Situ Gelation Study

Experimental details:

Total volume and the amount of imine **3** (experiments in Table S2) or amine **1** (experiments in Table S3) remained constant during the tests.

Table S2. Results of *in situ* gelation in propan-1-ol.

Molar ratio (amine **1** : aldehyde **2**)	3 : 1	2 : 1	1 : 1	1 : 2	1 : 3
Gelation tests (after a heat/cool cycle)	P	P	**G**	**G**	**G**
Weight/volume percentage of *in situ* formed imine **3**	2.00 %	2.00 %	2.00 %	2.00 %	2.00 %
Total weight/volume percentage	5.42 %	3.71 %	2.00 %	2.35 %	2.70 %

Table S3. Results of *in situ* gelation in propan-1-ol.

Molar ratio (amine **1** : aldehyde **2**)	1 : 0	1 : 0.2	1 : 0.4	1 : 0.6	1 : 0.8	1 : 0.9	1 : 1	1: 2	1 : 3
Gelation tests (after a heat/cool cycle)	S	S	P	P	P	**pG**	**G**	**G**	**G**
Weight/volume percentage of *in situ* formed imine **3**	0 %	0.40 %	0.80 %	1.20 %	1.60 %	1.80 %	2.00 %	2.00 %	2.00 %
Total weight/volume percentage	1.71 %	1.77 %	1.82 %	1.88 %	1.94 %	1.97 %	2.00 %	2.35 %	2.70 %

Note: S – solution upon cooling, P – partly precipitate upon cooling, pG – partial gel, G – gel.

Drug Release Experiments

Figure S12. Schematic image of slow-release and acid-induced release of a drug.

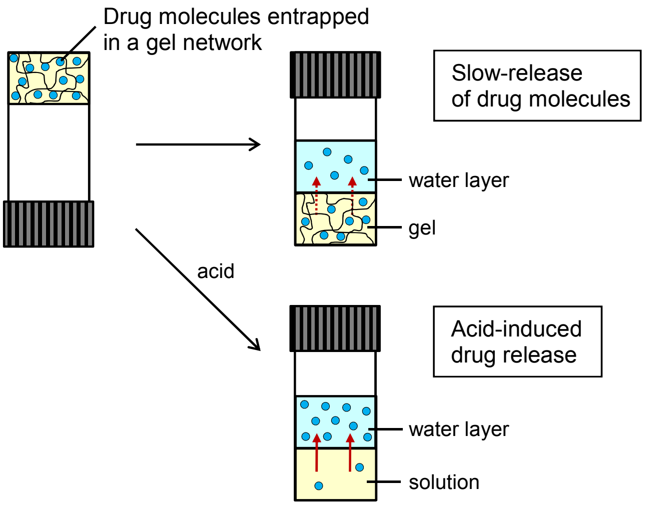

Experimental details:

The gels of imine **3** and pyrazinecarboxamide (2.8% w/v), prepared in a 1:1 ratio in 0.5 mL of pentan-1-ol (n_{PC} = 0.0195 mmol), were stabilised overnight. Then water (0.5 mL) either without or with *p*-toluenesulfonic acid (0.0053 mmol) was added. The samples stayed without any shaking or other type of disturbance. Water layers (0.4 mL) were separated off at certain times (after 0.5, 1, 2, 4 and 24 h), and after solvent evaporation in the open air, solid residues were dissolved in D_2O (0.6 mL) and analysed by NMR with succinic acid (0.0042 mmol) as an internal standard. As control experiments (A and B), pyrazinecarboxamide (0.0195 mmol) was dissolved in pentan-1-ol (0.5 mL), and the samples were treated in the same way as the gel samples (adding of 0.5 mL of water either without or with 0.0053 mmol of *p*-toluenesulfonic acid, and then analysed by NMR with 0.0085 mmol of succinic acid as an internal standard). To check the drug release under non-calm conditions, the samples were treated by ultrasonic for 10 min and after one hour of standing without any additional disturbance, the water layers were analysed by NMR in the same way like in the other drug release experiments. The percentage of the released drug was calculated from the peak area of drug signals of a sample to the peak area of drug signals of a reference sample which was prepared by dissolving pyrazine-carboxamide (0.0195 mmol) in 0.6 mL of D_2O with succinic acid (0.0085 mmol) as an internal standard. Results are summarised in Table S4.

Table S4 Results of drug release experiment.

	Peak area of drug signals I	II	III	Peak area calculated for 0.0085 mmol of succinic acid			Peak area calculated for the whole sample (0.4 mL -> 0.5 mL)			Percent of released drug			Average %
Reference	1.7883	1.8702	1.8686	a			b = 5/4 x a			c = b/reference			
**Control experiment A** (neutral conditions)				(used 0.0085 mmol of succinic acid)									
0.5 h	0.5856	0.6191	0.6165	0.5856	0.6191	0.6165	0.7320	0.7739	0.7706	40.9327	41.3793	41.2408	**41.18**
1 h	0.6023	0.6329	0.6350	0.6023	0.6329	0.6350	0.7529	0.7911	0.7938	42.1000	42.3016	42.4783	**42.29**
2 h	0.6341	0.6739	0.6747	0.6341	0.6739	0.6747	0.7926	0.8424	0.8434	44.3228	45.0420	45.1341	**44.83**
4 h	0.7315	0.7636	0.7671	0.7315	0.7636	0.7671	0.9144	0.9545	0.9589	51.1310	51.0373	51.3152	**51.16**
24 h	0.8082	0.8333	0.8265	0.8082	0.8333	0.8265	1.0103	1.0417	1.0332	56.4922	55.6973	55.2887	**55.83**
shaking	0.9410	1.0267	1.0382	0.9410	1.0267	1.0382	1.1763	1.2834	1.2978	65.7748	68.6223	69.4504	**67.95**
**Control experiment B** (acidic conditions)				(used 0.0085 mmol of succinic acid)									
0.5 h	0.5836	0.6164	0.6251	0.5836	0.6164	0.6251	0.7295	0.7706	0.7814	40.7901	41.2015	41.8147	**41.27**
1 h	0.6676	0.7042	0.7009	0.6676	0.7042	0.7009	0.8345	0.8802	0.8762	46.6616	47.0645	46.8880	**46.87**
2 h	0.6952	0.7307	0.7309	0.6952	0.7307	0.7309	0.8690	0.9134	0.9137	48.5908	48.8370	48.8949	**48.77**
4 h	0.8197	0.8597	0.8497	0.8197	0.8597	0.8497	1.0247	1.0746	1.0622	57.2974	57.4591	56.8420	**57.20**
24 h	0.8950	0.9400	0.9397	0.8950	0.9400	0.9397	1.1187	1.1750	1.1747	62.5566	62.8248	62.8626	**62.75**
shaking	0.9908	1.0546	1.0570	0.9908	1.0546	1.0570	1.2386	1.3182	1.3212	69.2585	70.4844	70.7053	**70.15**
**Gel** (neutral conditions)				(used 0.0042 mmol of succinic acid)									
0.5 h	0.7879	0.8119	0.8163	0.3940	0.4060	0.4082	0.4924	0.5074	0.5102	27.5366	27.1328	27.3032	**27.32**
1 h	0.9801	1.0548	1.0446	0.4901	0.5274	0.5223	0.6126	0.6593	0.6529	34.2539	35.2502	34.9393	**34.81**
2 h	1.0862	1.2139	1.1787	0.5431	0.6070	0.5894	0.6789	0.7587	0.7367	37.9620	40.5672	39.4246	**39.32**
4 h	1.2233	1.3161	1.3072	0.6117	0.6580	0.6536	0.7646	0.8225	0.8170	42.7536	43.9809	43.7226	**43.49**
24 h	1.3878	1.4876	1.4135	0.6939	0.7438	0.7068	0.8674	0.9298	0.8834	48.5028	49.7139	47.2780	**48.50**
shaking	1.5848	1.6963	1.7025	0.7924	0.8482	0.8513	0.9905	1.0602	1.0641	55.3878	56.6885	56.9444	**56.34**
**Gel** (acidic conditions)				(used 0.0042 mmol of succinic acid)									
0.5 h	1.1465	1.2065	1.2110	0.5733	0.6033	0.6055	0.7166	0.7541	0.7569	40.0695	40.3199	40.5049	**40.30**
1 h	1.2896	1.3815	1.3923	0.6448	0.6908	0.6962	0.8060	0.8634	0.8702	45.0707	46.1682	46.5690	**45.94**
2 h	1.3840	14553	1.4602	0.6920	0.7277	0.7301	0.8650	0.9096	0.9126	48.3700	48.6345	48.8400	**48.61**
4 h	1.5483	1.6310	1.6777	0.7742	0.8155	0.8389	0.9677	1.0194	1.0486	54.1121	54.5062	56.1149	**54.91**
24 h	1.7223	1.8358	1.8598	0.8612	0.9179	0.9299	1.0764	1.1474	1.1624	60.1933	61.3504	62.2057	**61.25**
shaking	1.9434	2.0451	2.0747	0.9717	1.0226	1.0374	1.2146	1.2782	1.2967	67.9207	68.3450	69.3935	**68.55**

Figure S12. ^{1}H-NMR spectra of drug release experiment in D_2O after 0.5, 1, 2, 4 and 24 h: (**a**) control experiment A (under neutral conditions); (**b**) control experiment B (under acidic conditions); (**c**) drug release from the gel under neutral conditions; (**d**) drug release from the gel under acidic conditions.

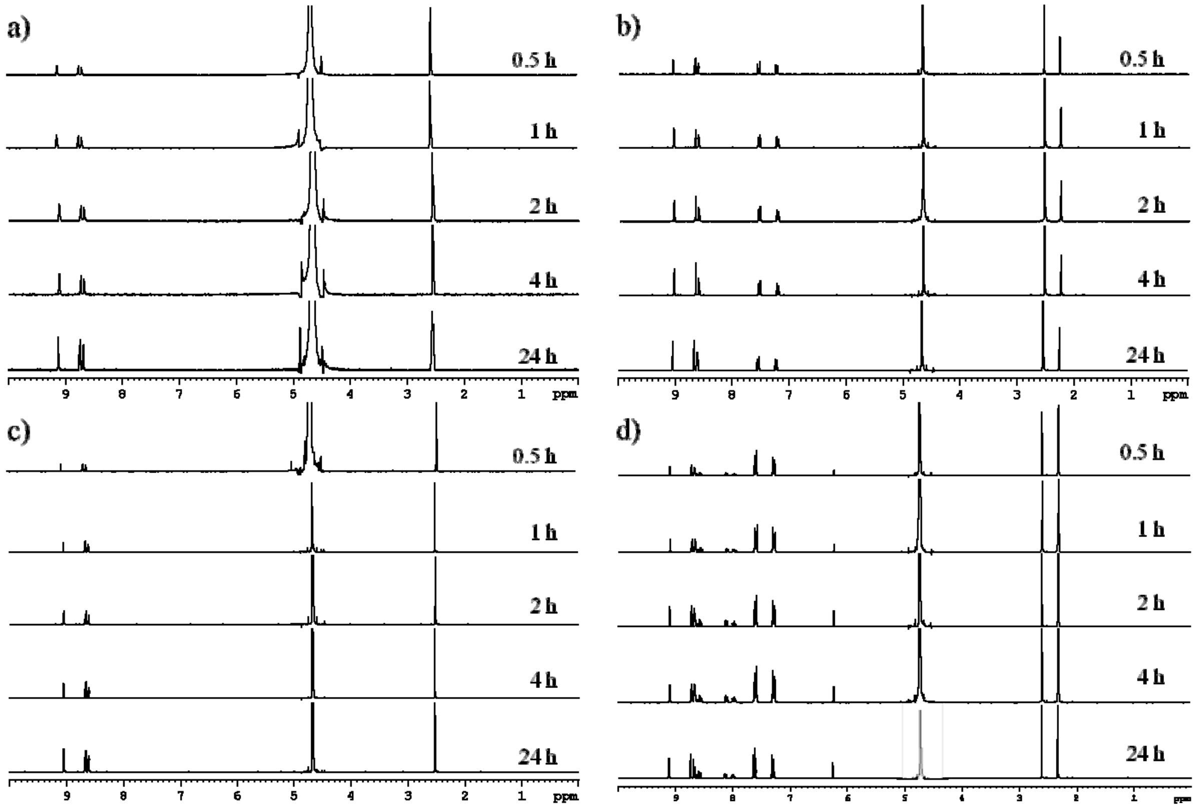

Supplement: Supplementary file 1 [file molecules-18-03745-s001.pdf]
